# Telehealth Movement-to-Music With Arm-Based Sprint-Intensity Interval Training to Improve Cardiometabolic Health and Cardiorespiratory Fitness in Children With Cerebral Palsy: Protocol for a Pilot Randomized Controlled Trial

**DOI:** 10.2196/56499

**Published:** 2024-03-05

**Authors:** Byron Lai, Robert A Oster, Drew Davis, Larsen Bright, Gordon Fisher, Jereme Wilroy, Yumi Kim, Raven Young, Ashley Wright, Tanvee Sinha, James H Rimmer

**Affiliations:** 1 Division of Pediatric Rehabilitation Medicine Department of Pediatrics University of Alabama at Birmingham Birmingham, AL United States; 2 Division of Preventive Medicine Department of Medicine University of Alabama at Birmingham Birmingham, AL United States; 3 Department of Human Studies University of Alabama at Birmingham Birmingham, AL United States; 4 Department of Physical Medicine and Rehabilitation University of Alabama at Birmingham Birmingham, AL United States; 5 Dean's Office School of Health Professions National Center on Health, Physical Activity and Disability Birmingham, AL United States

**Keywords:** disability, high-intensity, interval training, pediatrics, physical activity, telehealth

## Abstract

**Background:**

Children with mobility disabilities, including those with cerebral palsy, have limited options and limited time to exercise to manage their cardiometabolic health and cardiorespiratory fitness. Regular cardiovascular exercise during childhood is a critical health behavior for preventing health decline in adulthood. Thus, there is an urgent need for accessible, age-appropriate, convenient exercise modalities in this group. Sprint-intensity interval training (SIT), combined with telehealth procedures, may be ideal for children with disabilities. SIT includes repetitive bouts of maximal exercise effort combined with rest periods, which can be effective in eliciting comparable results to moderate-exercise training with very short training durations.

**Objective:**

This phase 1 pilot feasibility randomized controlled trial aims to investigate the potential effects of a 12-week SIT program on indicators of cardiorespiratory fitness and cardiometabolic health among children with cerebral palsy. An ancillary aim is to evaluate the feasibility of the program through several process feasibility metrics.

**Methods:**

This study uses a 2-armed parallel group design. A total of 50 physically inactive children with cerebral palsy (aged 6-17 years) will be randomly allocated into 1 of 2 groups: a 12-week SIT or a waitlist control group that continues habitual activity for 12 weeks. The SIT prescription includes 3 tele-supervised sessions per week with 30 repeated sequences of 4 seconds of maximal arm exercise, with active recovery, warm-up, and cooldown periods (for an approximately 20-minute total session). SIT includes guided videos with child-themed arm routines and music. The exercise sessions will be remotely supervised through a web-based videoconference application and include safety monitoring equipment. Outcomes are measured at pre- and postintervention (weeks 0 and 13, respectively). Health outcome measures include peak oxygen consumption (VO_2_ peak), measured by a graded exercise test; high-sensitivity C-reactive protein and blood insulin, hemoglobin A_1c_, triglycerides, and cholesterol using a finger stick dried blood spot test; blood pressure, using a sphygmomanometer; and body composition (total mass, total lean mass, tissue % lean, and tissue % fat) using dual x-ray absorptiometry. Feasibility will be evaluated by the following metrics: adverse events or problems experienced throughout the intervention related to participant safety; perceived enjoyment; and recruitment, enrollment, and attrition rates.

**Results:**

Recruitment procedures started in November 2023. All data are anticipated to be collected by February 2025. Full trial results are anticipated to be analyzed and submitted for publication by March 2025. Secondary analyses of data will be subsequently published.

**Conclusions:**

This trial tests an accessible and low-cost exercise program that leverages principles of high-intensity exercise to provide a convenient program for children with physical disabilities. Knowledge obtained from this study will inform the development of a larger trial for improving the cardiometabolic health, cardiorespiratory fitness, and well-being of children with physical disabilities.

**Trial Registration:**

ClinicalTrials.gov NCT05619211; https://clinicaltrials.gov/study/NCT05619211

**International Registered Report Identifier (IRRID):**

DERR1-10.2196/56499

## Introduction

### Overview of Cerebral Palsy

Cerebral palsy (CP) is a clinical syndrome that results from a developmental brain injury and is prevalent in approximately 1 million people in the United States and 23 million people worldwide [1]. CP is characterized by disorders of movement and posture due to nonprogressive disturbances of the fetal or developing brain, which is often accompanied by disturbances of sensation, perception, cognition, communication, and behavior [2]. Secondary neurological conditions such as epilepsy, along with other musculoskeletal issues, are also found among people who are diagnosed with CP [2]. Recent innovations in treatments and technology have increased the survival rates of people with CP, resulting in a steadily growing population of adults with CP [3]. Health professionals can now focus on how to help empower young people with CP to live well with a disability, amid a variety of health conditions and socioecological challenges they face with community engagement in adulthood [4,5].

### Adulthood and Cardiometabolic Risk

The transitory age between childhood and adulthood is a critical stage for health prevention interventions. As children with CP age into their 20s, they have a substantially increased risk of cardiovascular disease (CVD), related conditions, metabolic syndrome [6], and a 3-fold increased risk of CVD mortality compared to the general population [7-12]. The risk for these cardiometabolic diseases can be lowered by participation in regular moderate-intensity exercise [13-15]. However, recent studies demonstrate that children with CP do not engage in sufficient levels of exercise to obtain health-enhancing benefits, and these levels of exercise participation are far lower than those observed among children without CP [16-18]. Children with CP who exercise regularly are twice as likely to exercise as adults [16]. Consequently, there is a need to identify interventions that promote the early adoption of exercise as a behavior for preventing disease onset, particularly among children with CP who have a mobility disability and have the greatest risk of cardiometabolic disease [19].

### Limited Aerobic Exercise Options for Mobility Disability

Conventional exercises that are known to improve cardiorespiratory fitness (eg, walking, running, and cycling) generally require long durations of training that [20] are unsuitable for a large majority of children with CP. As children transition into adulthood, approximately 27% will be unable to walk, 35% will experience decreased walking ability, and 9% will lose their ability to walk [21,22]. Children and adults with CP often experience secondary conditions, such as hemiparesis, fatigue, impaired balance, and joint pain, and these factors impede their mobility to walk or perform types of exercise (eg, cycle) for prolonged periods [22-25]. A recent scoping review demonstrated that a top research priority in the field of disability and exercise is to develop aerobic exercises that are inclusive of wheelchair users [26]. The standard options for exercise training methods require leg work or full body involvement. Aside from arm-ergometry or wheelchair propulsion, there are limited aerobic exercise modalities that are efficacious or effective for people who require seated training, particularly modalities that accommodate hemiparesis [26]. Of note, no randomized controlled trial of exercise has demonstrated a clinically meaningful improvement in cardiorespiratory risk factors in children with CP, likely due to a lack of a sufficient exercise dose [26].

### Limited Generalizability of Research Interventions

A second notable limitation of published exercise interventions for CP is low rates of both recruitment and participation [26,27]. Despite over 30 years of research, including 49 published randomized controlled trials, the average sample size for a study was 30 [26]. Most participants were ambulatory, and wheelchair users were typically excluded from participation. These issues hinder the generalizability and transferability of study findings [26-30]. Moreover, low levels of participation in exercise trials were speculated to be due to the following barriers: (1) in-person supervision by a rehabilitation professional or exercise specialist, (2) on-site training at a laboratory or fitness facility, and (3) costly, specialized equipment [26,27]. The identification of an effective evidence-based modality that is inclusive of wheelchair users will require a protocol that can be replicated in a large trial that can provide confirmatory study findings.

### Rationale for Sprint-Intensity Arm Exercise

High-intensity interval training (HIIT) has been studied as an effective dose of exercise that has a moderate to large effect on cardiorespiratory fitness and cardiometabolic health while requiring a lower duration of training compared with continuous moderate-intensity exercise [31,32]. Sprint-intensity interval training (SIT) is a form of HIIT that also emphasizes short bouts of exercise with maximum effort at or above a power output associated with a peak oxygen consumption (VO_2_ peak) [33]. A meta-analysis [34] found that SIT has a moderate-to-large effect on VO_2_ peak that is similar to both moderate-intensity [35,36] and HIIT exercise [33]. Regarding the mechanism, SIT is believed to use anaerobic energy pathways of type IIB muscle fibers, the fast-twitch glycolytic pathways, which are replenished through oxidative metabolism [37], primarily from the kidneys [38]. In other words, extremely short bouts of exercise at maximal intensity can be sustained through the use of lower-intensity aerobic energy pathways. Specifically, higher exercise intensities result in increased renal hypoxemia and, thus, greater production of the hormone erythropoietin. Erythropoietin initiates the production of red blood cells that enhance oxygen use of performing muscles [39] and, thus, VO_2_ peak. SIT may further increase VO_2_ peak through mitochondrial biogenesis and capillary density [37]. A higher VO_2_ peak is linked with reduced risk for cardiometabolic disease [36,40], which is relevant among children with CP who generally have low levels of cardiorespiratory fitness [41]. HIIT with a treadmill has been found to be safe and effective in improving VO_2_ peak among ambulatory children with CP [42]. To the best of our knowledge, no randomized controlled efficacy or effectiveness trial has examined a wheelchair-accessible SIT program among children with CP [26,27]. SIT with arm-cycling results in greater gains in VO_2_ peak versus leg-cycling (eg, 52% vs 6% increase in VO_2_ peak, respectively) [43], likely due to a higher proportion of fast muscle fibers in the arms [43], and this information holds great value for nonambulatory children who can use their arms for exercise.

### Telehealth Interventions Foster Enrollment and Attendance

To address low participation, telehealth interventions are especially promising because they remove many of the geographic, environmental, and financial burdens associated with traveling and paying for community programs and classes [44]. Telehealth interventions are especially helpful in delivering health services to children because of reduced burden on the caregiver, who would otherwise have to miss work or other commitments to travel or allocate transportation for the child’s appointment [44]. Transportation and time are substantial barriers to exercise participation among people with disabilities [5]. Studies have shown that overall ratings of caregivers toward telehealth appointments are highly positive, and most view telemedicine consultations as at least as effective as those on-site; caregivers had modest reductions in out-of-pocket costs and less missed work costs [45]. In addition, telehealth programs are believed to enhance adherence by providing a sense of accountability, which is created by performance monitoring and social bonds with trusted professionals, as explained by the Supportive Accountability Theory [46]. Home-based exercise programs that incorporate telecommunications and monitoring have achieved the largest sample sizes of people with disabilities [26,27,47]. It is important to note that telehealth interventions that aim to promote strong attendance to an exercise regimen will require remote behavioral coaching strategies to maintain participant engagement. One meta-analysis found that exercise interventions that include behavioral techniques have stronger effects on exercise behavior than those that do not have such techniques [48].

### Study Rationale and Purposes

In consideration of exercise barriers (lack of time and transportation) and facilitators of engagement among children (enjoyable exercises and music), this study aims to test a program among children with CP that incorporates (1) age-appropriate prerecorded videos with movement-to-music exercises that are based on child-appropriate themes (eg, superheroes, sports, and pop music), (2) replicable cloud-based telemonitoring procedures, (3) behavioral tele-physical education supervision during exercise, and (4) SIT adapted for varying arm abilities.

### Aims

This study has 3 aims, described in following sections.

#### Aim 1: Examine the Effects of a 12-Week Home-Based SIT Program on Cardiorespiratory Fitness (VO_2_ Peak) Compared to a Waitlist Control Among 50 Children With CP

This study will compare pre-post changes in cardiorespiratory fitness between 2 study groups. One group will receive 12 weeks of home-based SIT (n=25), and the second group, waitlist control (WC; n=25), will resume their normal daily activities for 12 weeks. VO_2_ peak is the cardiorespiratory outcome of interest, which will be assessed by a graded exercise test using an arm ergometer. We hypothesized that the immediate start group would achieve greater improvements in VO_2_ peak versus WC.

#### Aim 2: Explore the Effect Estimates of SIT on Cardiometabolic Risk Indicators Versus WC

Cardiometabolic outcomes will include high-sensitivity C-reactive protein (hsCRP) and blood insulin, hemoglobin A_1c_ (HbA_1c_), triglycerides, and cholesterol using a finger stick dried blood spot test, and blood pressure using a sphygmomanometer. Additionally, body composition (total mass, total lean mass, tissue % lean, and tissue % fat) will be measured using dual x-ray absorptiometry (DXA). We hypothesized that arm-based SIT would demonstrate greater improvements in outcomes versus WC.

#### Aim 3: Evaluate the Feasibility of Arm-SIT Among Children With CP

Feasibility will be measured by process feasibility metrics [49] to inform a larger efficacy or effectiveness trial. Variables will include (1) intervention adherence (class attendance and video minutes through cloud-based analytics), (2) safety (adverse events or problems experienced throughout the intervention related to participant safety), (3) perceived enjoyment, and (4) engagement (recruitment, enrollment, and attrition rates).

## Methods

### Study Design and Overview

This phase 1, pilot feasibility randomized controlled trial will include a 2-armed parallel group design to examine the preliminary effects of 12 weeks of arm-based SIT on cardiorespiratory fitness compared to a WC that undergoes habitual activities for 12 weeks. The project will include 50 children with CP (n=25 per arm) and caregivers (n=50 parent-child dyads). One caregiver is required to participate by supporting their child’s safety through the intervention and managing the child’s exercise schedule. Participants will come to the laboratory for 2 total visits (baseline and week 13) to complete the data collection procedures. Participants will perform the intervention at home, with supervision by a research staff member through telecommunications.

### Participants

Textbox 1 contains eligibility criteria for child participants.

Contraindications were informed by guidelines that were established by the American College of Sports Medicine [50] and the Pediatric Physical Medicine and Rehabilitation physician of the study team (DD).

Eligible caregivers will include parents or legal guardians of the child, who can commit sufficient time to support the child in their roles for the study and communicate in English. Caregivers who have complete blindness or deafness will be excluded from participation.

**Eligibility criteria for child participants.**

**Inclusion criteria**

- medical diagnosis of cerebral palsy, as determined by the International Classification of Diseases–Tenth Revision codes
- age 6-17 years
- Gross Motor Function Classification System level 1 to 3 (as determined through participant screening, explained in the Screening and Recruitment section)
- medical clearance from a physician to participate in high-intensity exercise (using the attached medical screening form and explained in the intervention safety, monitoring, and response plan)
- access to a Wi-Fi internet connection in the home through a mobile phone or tablet computer
- a *z* score of ≤–3, indicative of high risk for fracture that could occur from torsion of spine

**Exclusion criteria**

- physically active (defined as >150 minutes per week of self-reported moderate to vigorous intensity exercise in a typical week)
- cannot use their arms for exercise
- Gross Motor Function Classification level of 4 to 5
- complete blindness or deafness
- pregnant
- uses a pacemaker
- has not been seen by a physician within the previous 12 months of their baseline visit
- uses a g-tube
- any past history of a contraindication to exercise testing: ischemia; myocardial infarction or other acute cardiac event; unstable angina; uncontrolled cardiac dysrhythmias; aortic stenosis; heart failure; pulmonary embolus or pulmonary infarction; myocarditis or pericarditis; aneurysm; low bone-mineral density of the spine (a *z* score of ≤–3, indicative of high risk for fracture that could occur from torsion of spine, determined at baseline data collection through the dual x-ray absorptiometry scan)

### Participant Roles

Both the child and caregiver are considered participants in the study, and both will sign the informed consent or assent document.

The child’s role in the study is to exercise, following along with exercise videos, 3 times per week for 12 weeks while being supervised through a videoconference web platform by a telecoach.

The caregiver’s roles in the study are to (1) attend each videoconference exercise session with the child, (2) schedule and manage the child’s exercise schedule, (3) support the safety of the child, and (4) achieve cardiopulmonary resuscitation (CPR) and automated external defibrillator (AED) certification through the Red Cross (certified by the principal investigator, BL, who is certified as a Red Cross CPR/AED instructor).

### Screening and Recruitment

Screening for this study will occur in 3 phases. First, contact lists are screened for a medical diagnosis of CP based on the International Classification of Diseases, Tenth Revision (ICD-10) codes. Second, during the initial recruitment contact with a caregiver, research staff will screen participants based on the study eligibility criteria. As noted previously, to enhance intervention safety, this study aims to enroll children with CP who have only mild to moderate mobility disability and have a low risk of adverse events from exercise. Therefore, the initial recruitment contact through telephone will include the Gross Motor Function Classification System (GMFCS) level [51], using the GMFCS Family Report Questionnaire [52-54]. This assessment will be asked over the phone to avoid wasting participants’ time arriving at the laboratory and learning that they do not qualify for the study. Third, a physician (study physician or participants’ physicians) will review the patient’s medical record and screen the participant for contraindications to exercise testing and risks for participation in high-intensity exercise (based on the Medical Clearance Form, Multimedia Appendix 1). In summary, if an absolute contraindication is identified, the participant will be excluded or withdrawn from the study. If no absolute contraindication is identified, the physician will identify major and minor risks based on the latest information within the patient’s medical record and then decide based on major and minor risks to clear the participant for high-intensity exercise training at home. To ensure that record review information is relevant, if the patient has not been seen in the past year, they will have to be seen before obtaining medical clearance and participating in the study.

Candidates will be prescreened and recruited primarily from medical and billing records from the Children’s Hospital of Alabama. Medical and billing record databases will be prescreened for potentially eligible patients by patient diagnoses codes (ICD-10, Clinical Modification) related to CP. Contact lists will be generated from the databases. Specific methods of recruitment will include word of mouth and flyers, physician, and staff referrals, mailouts, and phone calls.

### Randomization and Clinical Trial Considerations

Parent-child dyads will be randomized into 1 of 2 groups: arm-based SIT or WC (n=25 per group) with a 1:1 allocation ratio using a permuted block randomization approach. The randomization sequence was generated by the project statistician (RAO) using a computer-generated randomized permuted block design (SAS software, version 9.4; SAS Institute). The project statistician sent the randomization sequence to the database manager (RY) during the study preparation phase. The database manager will unfold the randomization sequence for successfully enrolled participants into 1 of the 2 groups. Study outcomes will be assessed by a research assistant, who will be blinded to group allocation (single-blinded trial design). Recruitment will occur primarily through the Children’s Hospital of Alabama. All other activities will be conducted at the Human Performance Laboratory in the Wellness Health and Research Facility at the University of Alabama at Birmingham.

### Equipment and Telehealth Monitoring Platform

**Figure 1:**
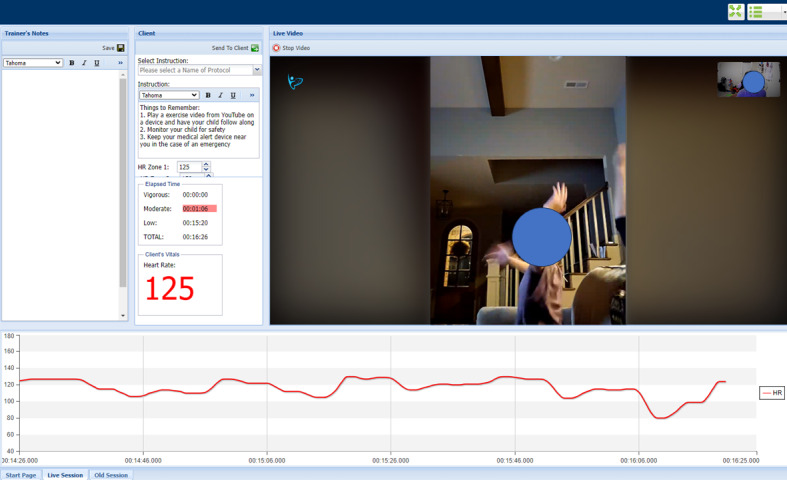
Telecoach view of an exercise session with a participant through the TeleRehab app.

The study will use a web-based platform with physiologic devices to support remote telehealth supervision of the intervention. The platform, referred to as TeleRehab, includes an Android app that is installed on a computer tablet with Bluetooth capability, which sends data and allows 2-way communications to a secure web server. This setup allows a user on an Android computer tablet to communicate in real time with a member of the research team on a desktop computer. Communication features include videoconference and SMS text messaging. Heart rate data are recorded and transmitted for real-time view by both the user and research staff using a heart rate device (Polar Sense). The heart rate device was placed on a headband and worn on the head to avoid disruption caused by the rapid and vigorous arm movements required for the exercise intervention. Based on internal testing among research staff (nonpublished findings), placement on the head may result in more accurate readings during intervention exercise than when the device was worn on the forearm or upper arm. The device instructions from the manufacturer state that the Polar Sense can be worn on the head, forearm, or upper arm. The TeleRehab app system was an upgraded version of one used previously in a telemonitored feasibility exercise study among people with spinal cord injury [55]. A demonstration of an exercise session in the TeleRehab web portal, as viewed by a telecoach, is displayed in Figure 1. Additional equipment to support the safety of the intervention exercise sessions will include: (1) a blood pressure monitor (Omron HEM-7200/BP7200, 5 Series); (2) a medical alert device (Monitored Medical Alert System), a necklace-worn device that allows 2-way communication between the user and the American District Telegraph (ADT) emergency response team, who is available at all hours of the day to provide remote support for obtaining local emergency response to the user; and (3) a defibrillator (Philips HeartStart OnSite AED Home). Participants will be trained to use all devices at baseline data collection.

### Procedures

Participants in the immediate start group will complete a 12-week exercise intervention with 2 data collection visits to the laboratory (pre- and postintervention; weeks 0 and 13, respectively). Participants in the control group will wait 12 weeks before starting the intervention, and they will also complete week 0 and week 13 data collection. Before arrival on a data collection visit, participants will be asked to fast for 10 to 12 hours overnight.

The first data collection visit (week 0) will include the following: (1) study briefing (purpose, procedures, roles, risks, and benefits), (2) informed consent and assent documentation, (3) study questionnaires, (4) cardiorespiratory fitness and cardiometabolic outcome measures, (5) Red Cross CPR and AED training for the caregiver, (6) ADT subscriber agreement form (to receive a medical alert device, shipped to the home from ADT), and (7) intervention or wait briefing (12-week prescription and equipment instructions).

The second data collection visit (week 13) will include (1) study questionnaires, (2) cardiorespiratory fitness and cardiometabolic outcome measures, and (3) debriefing (reviewing pre-post changes in outcomes) for the intervention group or intervention briefing for the control group.

### Measures

#### Questionnaires

The study will include a demographic questionnaire of participant characteristics (eg, age, sex, and BMI) and the Godin Leisure-Time Exercise Questionnaire (GLTEQ). The GLTEQ [56] is a 3-item self-report questionnaire that is used to measure physical activity participation. The GLTEQ asks participants to report the number of times that low, moderate, and vigorous intensity of physical activity was performed for longer than 15 minutes in a typical week. The numbers reported for moderate and vigorous exercise intensity are then multiplied by 7 and 9, respectively, and summed for a score, which is referred to as the health contribution score. A health contribution score of <24 is classified as physically inactive, whereas a score of ≥24 is considered physically active [57]. There is evidence to support the GLTEQ as a valid and reliable measure of physical activity among adults with multiple sclerosis [58] and adolescents [59].

#### Cardiometabolic Outcomes

##### Aim 1 Primary Outcome: Peak Oxygen Consumption (VO_2_ Peak)

The V0_2_ peak will be measured during a graded exercise test on an arm ergometer using open-circuit spirometry with a metabolic cart (TruOne, ParvoMedics). Arm ergometers are considered the gold-standard modality for exercise testing among people with disabilities who use wheelchairs or cannot run or cycle for prolonged periods [60,61]. Arm ergometry has been suggested as a valid and reliable method for measuring VO_2_ peak among children with CP with mild to moderate disability [62]. Before starting the test, participants rest for 3 minutes. They will then be instructed to maintain a pedaling cadence of 60 revolutions per minute. Resistance is increased every minute by 10 watts until the participant reaches volitional fatigue or achieves 3 of 5 criteria: age-predicted heart rate max of more than 85%; 17 or higher on the Borg rating of perceived exertion (RPE) 6-20 scale; respiratory exchange ratio of 1:1 or higher; plateau in oxygen consumption; volitional fatigue [63]; and subjective signs of exhaustion [64]. Heart rate and oxygen consumption will be measured continuously.

##### Aim 2 Outcomes: Indicators of Cardiometabolic Health

Cardiometabolic health will be measured through a dried blood spot test, a blood pressure cuff, and a DXA scan.

###### DXA Scan

DXA scan variables will include total mass, total lean mass, tissue % lean, and tissue % fat. Scans will be analyzed using CoreScan software (GE HealthCare).

###### Blood Pressure (mm Hg)

Blood pressure will be measured by a sphygmomanometer. Elevated blood pressure during childhood is associated with intermediate markers and hard outcomes of CVD in adulthood [65]. Small changes in blood pressure (–6.4 mm Hg systolic and –4 mm Hg diastolic pressure) can occur from endurance training interventions with durations of up to 12 weeks [66].

###### Dried Blood Spot Fingerstick Test

A dried blood spot fingerstick test (ZRT Laboratory) will be used to assess blood-related cardiometabolic profiles and include hsCRP, HbA_1C_, fasting insulin, triglycerides, and cholesterol (total, low-density lipoprotein [LDL], and high-density lipoprotein [HDL]). Dried blood spot tests will be shipped for analysis to the ZRT Laboratory [67]. The ZRT Laboratory blood spot test has been conducted with children [68,69] and has demonstrated excellent validity with venous serum samples (eg, hsCRP, *r*=0.99; fasting insulin, *r*=0.93; fasting triglycerides, *r*=0.95) [67,70].

*hsCRP (mg/L)*: C-reactive protein is a critical marker of inflammation that contributes to proinflammatory and prothrombotic elements of CVD risk. A single hsCRP measure is a strong predictor of myocardial infarction or coronary heart disease mortality, and several other diseases of the circulatory system in people without a history of such conditions [71]. Changes in hsCRP may occur from as early as 8-weeks of exercise [72].

*HbA_1c_ (mmol/mol)*: HbA_1c_ is a measure of red blood cell mean hemoglobin glycation over the previous 3 months. A 1-month exercise intervention without a dietary component can expect a small to moderate effect [73].

*Fasting Insulin (μIU/mL)*: High fasting insulin indicates the presence of insulin resistance, whether an individual shows glucose intolerance. Exercise interventions without a dietary component can expect a small beneficial change in fasting insulin levels after 1 month of training [73].

*Fasting Triglycerides (mg/dL)*: A triglyceride level >150 mg/dL, is supported as an indicator of CVD risk [74,75]. Exercise interventions without a dietary component can expect a small beneficial change in triglyceride levels following 1 month of training [73], even among people with normal triglyceride levels [76].

*Fasting Cholesterol (mg/dL)*: Abnormalities in the lipid profile, including high total cholesterol (TC), high LDL cholesterol, and low HDL cholesterol, are predictors of future CVD among young and middle-aged people [77,78]. Exercise interventions without a dietary component can expect a small effect after 1 month [73].

##### Aim 3 Intervention Feasibility

Feasibility will be measured using process metrics that will inform a larger trial [49]. Metrics will include adherence to the exercise prescription (measured through an attendance log managed by the telecoaches and TeleRehab cloud-analytics); technological issues; perceived enjoyment after completing the program will be assessed using the Physical Activity Enjoyment Scale (PACES) [79]; recruitment, enrollment, and attrition rates; and adverse events or problems experienced throughout the intervention.

### Home-Based Intervention

**Figure 2:**
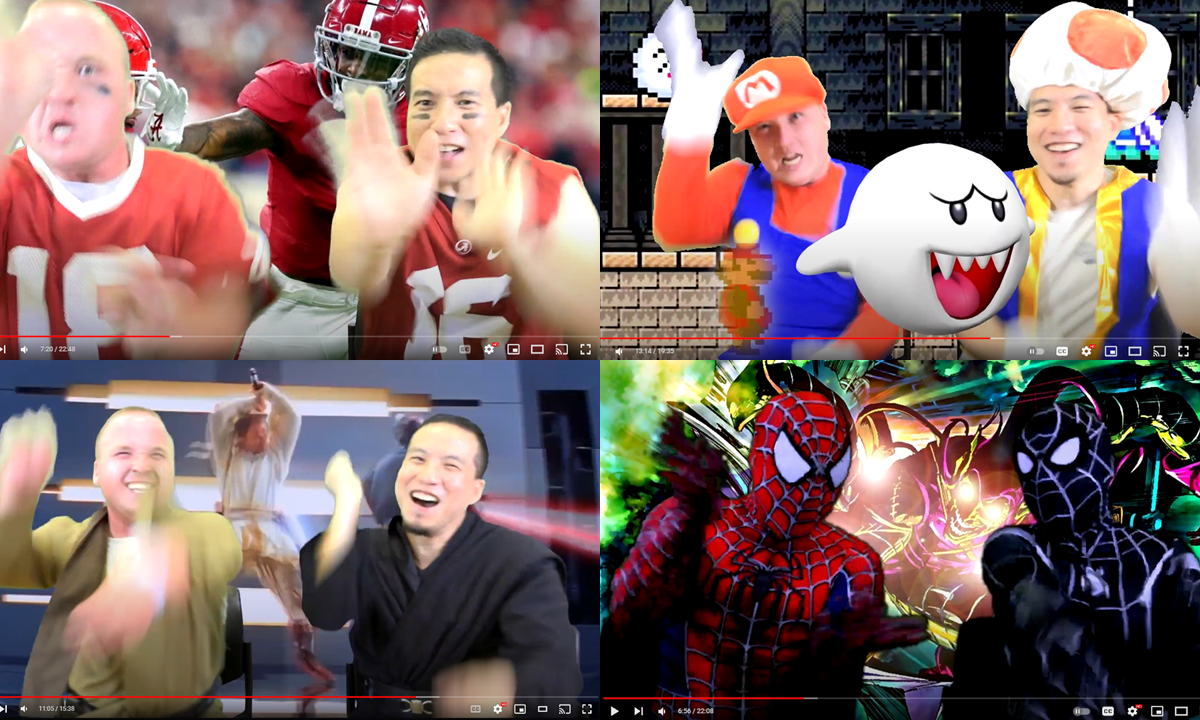
Exercise video themes guided by a disability exercise specialist and a young adult with cerebral palsy.

The SIT intervention will be performed by participants at home and include 3 sessions of exercise per week for a total of 12 weeks. Each SIT session will include 30 repeated sequences of 4 seconds of maximal arm exercise [38], supplemented with active recovery periods of low-intensity posterior shoulder movements to prevent overuse of anterior muscles. Recovery will be gradually reduced from 30 seconds in week 1, 24 seconds in weeks 2-4, to 15 seconds in weeks 5-12 [38]. The total session duration changes from 24 minutes in week 1 to 16.5 minutes in weeks 6-12. Included in the total session duration are a 5-minute warmup and a 2-minute cooldown. A similar leg exercise protocol was found to be effective for raising VO_2_ peak [38], and this dose may be optimal for achieving a high training volume while preventing fatigue due to muscle acidosis [38,80]. To achieve maximum intensity, participants are encouraged by the guidance in the videos to perform the 4-second bouts at their maximal effort. Regarding progression, resistance bands (low, medium, and heavy) will be gradually introduced into the exercises so that a participant will report a near maximum intensity (≥7 on the Borg RPE 0-10 scale; explained in detail in the next section) [81,82]. Children with CP from a home-based movement-to-music pilot intervention, from which this study protocol was based, reported that 20-minute videos would be ideal for their attention spans and busy school and care schedules [83]. Sessions will be guided by prerecorded videos that include an adapted exercise instructor (principal investigator BL) and a young adult actor with CP (LB; Figure 2).

Videos will include 5 themes that were requested by children with CP from our pilot: 3 themes related to superheroes, 1 theme for pop music, and 1 theme for sports. Each theme will include 3 sequential videos that coincide with the rest periods (1 video for week 1, 1 video for weeks 2-4, and 1 video for weeks 5-12), totaling 15 videos. Children with CP can choose to exercise with any themed video that corresponds to their exercise week. Videos include songs that relate to the video themes. Music tempo and volume coincide with sprint and recovery periods. Sprint training incorporates repetitive arm movements that relate to video themes (eg, Spiderman videos include web shooting, football, and arm running or passing). Participants are instructed by the 2 actors in the videos, who demonstrate and guide participants through the exercises. Videos include CP-specific adaptations to enhance engagement [83]: verbal cues, visualized movement adaptations for hemiparesis, slow and repetitive instructions, positive reinforcement, judgment-free atmosphere, and imagery. Videos will be archived within a YouTube playlist. The benefits of archiving videos within YouTube (as opposed to a custom-designed mobile or web application) are that videos are easily accessible on any device, many people are already familiar with YouTube, and this process can easily be replicated. Videos will include themes that are based on copyrighted material, which will only be used for research purposes to adhere to “fair use” for “education” and “research purposes” of the Fair Use Act Copyright Disclaimer, Section 107 of the Copyright Act 1976.

WC participants will be asked to maintain their habitual activity behavior for 12 weeks and then receive the SIT intervention. All participants will be asked to maintain their habitual diet and eating patterns.

### Intervention Development

The SIT intervention was adapted from a clinical adult exercise program, referred to as movement-to-music [84]. The program originated from a clinical efficacy trial that demonstrated improvements in lower extremity function and fatigue among adults with mobility disabilities [84]. The success of movement-to-music led to its implementation in larger confirmatory trials among adults with disabilities [85-87]. In contrast, a pilot feasibility trial found that several aspects of movement-to-music required modification to enhance participant engagement among children with CP who had mobility disabilities [69]. Findings from that study informed the development of the present SIT program.

### Telecoach and Fidelity

This study will test a high volume of supervised training to ensure children with CP achieve the desired intensity before testing a more scalable self-regulated protocol in a larger randomized controlled trial. All 3 of the weekly prescribed SIT sessions will be supervised through a Zoom (Zoom Video Communications) videoconference by a telecoach. The telecoach will support participants in maintaining maximal effort intensity by monitoring RPE and providing motivational support. Motivational strategies will include positive verbal encouragement to bolster exercise confidence (ie, self-efficacy) and performing the exercises with the participant to enhance learning through observation (ie, vicarious learning). These strategies are informed by social cognitive theory [88], and the research team has used these strategies in other trials [55,83,86,89]. To monitor exercise intensity, the telecoach will ask participants how hard they are working after every change in song (~5 minutes) throughout the session. If participants report <7 RPE, coaches or caregivers will provide positive verbal encouragement to enhance motivation. Post session, participants will report an overall RPE for the entire bout to determine the need to add resistance bands. RPE has been found to be effective in controlling vigorous exercise intensity in people with spinal cord injury [90]. Since caregiver knowledge and attitude are determinants of participation [91-93], they will be asked to help manage the child’s exercise schedule. The telecoach procedure was framed on supportive accountability theory: building a social relationship and bond with a trusted and knowledgeable health professional, with the inclusion of motivation strategies, can enhance intervention adherence [46].

### Analyses

#### Statistical Power

The chosen sample size of 50 participants permits at least 20 in each of the 2 study groups at 12-week follow-up after allowing for up to 20% dropout (in each arm). Power calculations were performed using nQuery (version 8.7) for aims 1 and 2, and SAS (version 9.4) for aim 3.

For aims 1 and 2, baseline measurements will be compared with those obtained in week 13. We obtained estimates of the SD for VO_2_ peak of 7.11 mL/kg/min [64], percent body fat (BF) of 8.5% [94], TC of 27.3 mg/dL [95], systolic blood pressure (SBP) of 14.0 mmHg [95], and diastolic blood pressure (DBP) of 11.7 mmHg [95]. With these assumptions and those of a 2-sided 2-group *t* test, an α of .05, and 20 participants per group, the study will have at least 80% power to detect between-group differences of at least 6.47 mL/kg/min in VO_2_ peak, 7.7% BF, 24.9 mg/dL in TC, 12.8 mmHg in SBP, and 10.7 mmHg in DBP as being statistically significant (at any time point); with the previous assumptions and those of a 2-sided paired *t* test, an α of .05, and 20 participants, we will have at least 80% power to detect within-group changes of at least 4.70 mL/kg/min in VO_2_ peak, 5.6% BF, 18.1 mg/dL in TC, 9.3 mmHg in SBP, and 7.8 mmHg in DBP as being statistically significant (between any 2 time points).

For aim 3, we anticipate adherence to the supervised exercise sessions of this study will be 75%, based on the 70% adherence found from our pilot, which had unsupervised sessions and videos that were not designed for adolescents. For an adherence rate of 75%, and assuming 40 participants in total, with 20 per study arm, complete the study, the corresponding exact binomial 95% CIs are 0.588-0.873) and 0.509-0.913.

#### Analyses

For all aims, data analyses will follow intent-to-treat principles. Descriptive statistics will be obtained and reported for study variables. The normality of data distribution of the continuous outcomes will first be confirmed using graphical techniques and tests of normality; continuous outcomes that deviate greatly from a normal distribution will be adjusted (eg, transformed) so that the data distribution approximates a normal distribution. All statistical tests will be 2-sided. Differences will be considered significant at *P*<.05. Statistical analysis will be performed using SAS software (version 9.4). Missing data that are not rectified through ongoing review of source documents may be managed with multiple imputations, and the influence of the missing data will be assessed with sensitivity analyses.

For aims 1 and 2, our primary method of analysis will be general linear mixed model techniques, such as mixed model repeated measures analyses, as there will be 2 study groups (SIT and WC) and 3 time points (baseline, 6 weeks, and 12 weeks). Post hoc analyses will be performed using the Tukey-Kramer multiple comparisons test. These models will allow us to assess the between-group effect, the within-group effect, and the group-by-time interaction. Covariates to be included in some models include age, sex, and GMFCS level. Analysis of categorical variables between groups will be performed using the chi-square test (or Fisher exact test, if needed). Pairwise correlations between study parameters will be assessed using Pearson (or Spearman, if needed) correlation analysis.

For aim 3, we will calculate adherence rates, adverse event rates, and other rates of interest and corresponding exact binomial 95% CIs. These rates will be calculated overall and stratified by the study group. Exploratory comparisons of rates between groups will be performed using the chi-square test (or Fisher exact test, if needed).

### Ethical Considerations

The protocol and informed consent and assent forms were approved by the University of Alabama at Birmingham Institutional Review Board for Human Use (IRB-300008913) on January 12, 2023. Prospective participants provide written informed consent or assent documentation before participation in the study. Consent and assent forms are completed on the baseline data collection visit.

Before the enrollment of participants, this study was approved by the institutional review board of the university. Written informed consent documentation will be obtained from all participants before their engagement in the study. Participants will receive an electronic gift card that will be loaded for US $500 for each of the data collections completed, for a total of up to US $1000. The rationale for this value is to account for the commitment and time required of both the child and caregiver to complete the study. No artificial intelligence software or program was used in the writing of this manuscript.

## Results

This study was approved by the university’s institutional review board on January 12, 2023. The study was funded and initiated in May 2023, and the first participant was enrolled in October 2023. As of this manuscript submission on February 7, 2024, a total of 11 people have been enrolled in the study. The study’s end date is April 30, 2025. The anticipated publication of the study findings will be in March 2025.

## Discussion

### Overview

This study will investigate the preliminary efficacy of a home-based, low-cost program for improving cardiorespiratory fitness and metabolic health that is child-appropriate for CP. A program that is short in duration, accessible for various physical abilities, and can be accessed anywhere through the internet could benefit this population. CP is a low-prevalence disability group that is often geographically isolated from suitable clinics, specialists, and adapted exercise services. Moreover, children with CP have limited options for exercise that are evidenced to improve their health [26,27] and have experienced lower participation in exercise since the outbreak of the COVID-19 pandemic [96].

### Strengths and Limitations

Considering that this program can be delivered entirely through an internet video cloud server, YouTube, it has the potential to be carried forward in a scale-up randomized controlled trial of exercise for children with CP. This is important, considering that the average sample size for a randomized controlled trial for children and youths with disabilities has been found to be 27 people, and one of the largest trials included 159 people [27], numbers that are certainly not representative of the diverse needs of children with CP [97]. Another strength of this study is the incorporation of child-appropriate themes and music. Music-based therapy has been found to have an effect on functional ability and goal attainment among children with CP [98]. Child-appropriate themes were requested by this age group and could enhance engagement in therapeutic exercise [83].

This is a preliminary study with a high focus on examining the safety of the intervention and its efficacy under carefully controlled conditions (supervised exercise sessions). Supervised training through telehealth communication provides a sense of accountability that enhances adherence to the intervention prescription [46]. Thus, this study assumes that participants will adhere strongly to the intervention exercise, and the research question is not whether they will perform the exercise; instead, the question focuses on how well they will enhance their health with strong attendance. Should this trial be successful, there will be a need to compare the effects of supervised exercise training versus self-regulated, asynchronous training, which will be the primary format in which prerecorded videos will be used in real-world settings. Supervised training is burdensome for both the research staff and participants.

An additional limitation of this study is the requirement for on-site data collection procedures, which warrants investigation. On-site visitations are difficult for children with CP and their caregivers due to difficulties with transportation and lack of time with busy school schedules, therapies, and daily activities [5,99]. There is a need to develop home-based data collection procedures that provide comparable results to those obtained from in-person testing. The blood spot test can be performed remotely by shipping blood spot test kits to the homes of children with CP. However, to the best of our knowledge, there is no home-based alternative method for measuring cardiorespiratory fitness or body composition imaging, particularly among people with mobility disabilities [20].

### Conclusions

Should the findings of this study suggest that the program can improve cardiorespiratory fitness or cardiometabolic health, the study may discover an innovative and, most importantly, scalable method of exercise intervention among children with physical disabilities.

## Appendix

Multimedia Appendix 1 Medical clearance screening form.

Multimedia Appendix 2 CONSORT-eHEALTH checklist (V 1.6.2).

Multimedia Appendix 3 Peer-review report from the National Institutes of Health (NIH).

## Abbreviations

ADT: American District Telegraph
AED: automated external defibrillator
BF: body fat
CP: cerebral palsy
CPR: cardiopulmonary resuscitation
CVD: cardiovascular disease
DBP: diastolic blood pressure
DXA: dual x-ray absorptiometry
GLTEQ: Godin Leisure-Time Exercise Questionnaire
GMFCS: Gross Motor Function Classification System
HbA_1c_: hemoglobin A_1c_
HDL: high-density lipoprotein
HIIT: high-intensity interval training
hsCRP: high-sensitivity C-reactive protein
ICD-10: International Classification of Diseases–Tenth Revision
LDL: low-density lipoprotein
PACES: Physical Activity Enjoyment Scale
RPE: rating of perceived exertion
SBP: systolic blood pressure
SIT: sprint-intensity interval training
TC: total cholesterol
VO_2_ peak: peak oxygen consumption
WC: waitlist control

## Data Availability

The data sets generated and analyzed during this study will be made publicly available at the completion of the study and can be obtained from the corresponding author on reasonable request.
