# Supplementary material for: Telehealth Movement-to-Music With Arm-Based Sprint-Intensity Interval Training to Improve Cardiometabolic Health and Cardiorespiratory Fitness in Children With Cerebral Palsy: Protocol for a Pilot Randomized Controlled Trial
Source: JMIR Res Protoc. 2024 Mar 5;13:e56499. doi: 10.2196/56499 (PMC10951837; doi:10.2196/56499)
Supplement: Multimedia Appendix 1 [file resprot_v13i1e56499_app1.docx]

**Research Study Medical Clearance – Screening Form**

*This form is provided to physicians to provide medical clearance for their patients to participate in a high-intensity home-exercise program.*

Your Name: _________________________

Patient Name: _______________________

Patient Age: _________________________

Primary Diagnosis: ____________________________________________

**a)** ABSOLUTE CONTRAINDICATION TO EXERCISE (if one of these is checked, the rest of the answers can be skipped, and the document signed and dated)

- A recent significant change in the resting electrocardiogram suggesting significant ischemia, recent myocardial infarction (within 2 days), or other acute cardiac event
- Unstable angina
- Uncontrolled cardiac dysrhythmias causing symptoms or hemodynamic compromise
- Symptomatic severe aortic stenosis
- Uncontrolled symptomatic heart failure
- Acute pulmonary embolus or pulmonary infarction
- Acute myocarditis or pericarditis
- Suspected or known dissecting aneurysm
- Acute systematic infection, accompanied by fever, body aches, or swollen lymph glands
- Low bone mineral density of the spine (z-score of <=/ -3) or high risk of fractures in spine and upper arms

**b)** Please Indicate If the Patient Has Any of the Following **MAJOR** Risk Factors:

- Coronary artery disease: _________________________
- Heart arrythmia or disease: _______________________
- A previous adverse cardiac event
- Non-sustained ventricular tachycardia
- Unexplained syncope
- Extreme left ventricular hypertrophy
- Primary electrical disease with no specific cause identified
- Electrical disorder (Long QT syndrome or catecholaminergic polymorphic ventricular tachycardia)
- Other: _____________________________________________

**c)** Please Indicate If the Patient Has Any of the Following **MINOR** Risk Factors (Circle the options below):

- Family history of sudden cardiac death
- Abnormal blood pressure response to exercise
- Left ventricular outflow tract obstruction
- The participant is a male
- Other: _____________________________________________

**d)** Please Make Your Determination Below

- **Cleared for High-Intensity Exercise**

(Patient has no Contraindication, and the Major and Minor risk factors do not outweigh the potential benefits of participation in high-intensity exercise)

- **Exclude**

(Patient has at least one CONTRAINDICATION and/or the Major and Minor risk factors outweigh the potential benefits of participation in high-intensity exercise)

Notes:

SIGNATURE: _________________________________________ DATE: ______________________

1. Liguori G, American College of Sports Medicine. ACSM's guidelines for exercise testing and prescription. Lippincott Williams & Wilkins; 2020 Dec 3.
2. Norrish G, Cantarutti N, Pissaridou E, Ridout DA, Limongelli G, Elliott PM, Kaski JP. Risk factors for sudden cardiac death in childhood hypertrophic cardiomyopathy: A systematic review and meta-analysis. European journal of preventive cardiology. 2017 Jul 1;24(11):1220-30.
3. Aro AL, Chugh SS. Prevention of sudden cardiac death in children and young adults. Progress in pediatric cardiology. 2017 Jun 1;45:37-42.
