# Supplementary material for: Telehealth Movement-to-Music With Arm-Based Sprint-Intensity Interval Training to Improve Cardiometabolic Health and Cardiorespiratory Fitness in Children With Cerebral Palsy: Protocol for a Pilot Randomized Controlled Trial
Source: JMIR Res Protoc. 2024 Mar 5;13:e56499. doi: 10.2196/56499 (PMC10951837; doi:10.2196/56499)
Supplement: Multimedia Appendix 2 [file resprot_v13i1e56499_app2.pdf]

CONSORT-EHEALTH Checklist V1.6.2 Report	Manuscript Number	56499
**(based on CONSORT-EHEALTH V1.6), available at [http://tinyurl.com/consort-ehealth-v1-6].**		
**Date completed**		
2/14/2022 14:57:14		
**by**		
Byron Lai		
TITLE		
**1a-i) Identify the mode of delivery in the title**		
**1a-ii) Non-web-based components or important co-interventions in title**		
home-based telehealth Movement-to-Music program		
**1a-iii) Primary condition or target group in the title**		
there are no cointerventions, this is not applicable		
ABSTRACT		
**1b-i) Key features/functionalities/components of the intervention and comparator in the METHODS section of the ABSTRACT**		
**1b-ii) Level of human involvement in the METHODS section of the ABSTRACT**		
M2M included videos that participants were asked to complete three times each week at home.		
**1b-iii) Open vs. closed, web-based (self-assessment) vs. face-to-face assessments in the METHODS section of the ABSTRACT**		
M2M included videos that participants were asked to complete three times each week at home		
**1b-iv) RESULTS section in abstract must contain use data**		
Participants were recruited from a Children's hospital		
**1b-v) CONCLUSIONS/DISCUSSION in abstract for negative trials**		
Mean adherence to the videos was 90% (44/49 minutes) in week 1; 83% in week two (56/68 minutes); 69% in week 3(45/65 minutes), and 43% in week 4 (40/95 minutes). Adherence to the coaching calls was 98% in week 1; week 2, 90%; week 3, 90%; and week 4, 86%.		
INTRODUCTION		
**2a-i) Problem and the type of system/solution**		
**2a-ii) Scientific background, rationale: What is known about the (type of) system**		
a need to identify programs that can easily be translated beyond clinical and research-supported settings to reach larger populations of people with CP.		
METHODS		
**3a) CONSORT: Description of trial design (such as parallel, factorial) including allocation ratio**		
I have no other questions or comments.		
**3b) CONSORT: Important changes to methods after trial commencement (such as eligibility criteria), with reasons**		
examine the preliminary efficacy of a youth-based adapted M2M intervention for increasing both activity and LTPA participation		
**3b-i) Bug fixes, Downtimes, Content Changes**		
**4a) CONSORT: Eligibility criteria for participants**		
This study was a parallel-arm pilot RCT		
**4a-i) Computer / Internet literacy**		
**4a-ii) Open vs. closed, web-based vs. face-to-face assessments:**		
ability to use a device that was capable of viewing Internet-video content		
**4a-iii) Information giving during recruitment**		
Participants were recruited via telephone through medical and billing records and physician referrals from the Children's Hospital.		
**4b) CONSORT: Settings and locations where the data were collected**		
Due to COVID-19 University related delays, the length of the intervention was shortened to 4 months from the originally proposed 8 months, prior to conducting the trial (approved by the study sponsor).		
**4b-i) Report if outcomes were (self-)assessed through online questionnaires**		
**4b-ii) Report how institutional affiliations are displayed**		
They had two options to complete the assessments: 1) physically mailed documents that were in a large envelope containing the informed consent document and all outcome measures or questionnaires; or 2) signature of a digital consent document through a secure electronic database (REDCap) and completion of the questionnaires through phone calls with research staff.		
**5) CONSORT: Describe the interventions for each group with sufficient details to allow replication, including how and when they were actually administered**		
**5-i) Mention names, credential, affiliations of the developers, sponsors, and owners**		
**5-ii) Describe the history/development process**		
The present M2M intervention included the early adoption phase (first 4 weeks) of a larger 12-week M2M program for adults (#5R01HD085186-02)		
**5-iii) Revisions and updating**		
The present M2M intervention included the early adoption phase (first 4 weeks) of a larger 12-week M2M program for adults (#5R01HD085186-02)		
**5-iv) Quality assurance methods**		
This is not applicable to the study.		
**5-v) Ensure replicability by publishing the source code, and/or providing screenshots/screen-capture video, and/or providing flowcharts of the algorithms used**		
Children were instructed to complete the questionnaires with the assistance of a caregiver.		
**5-vi) Digital preservation**		
This is not applicable to the study.		
**5-vii) Access**		
This is not applicable to the study.		
**5-viii) Mode of delivery, features/functionalities/components of the intervention and comparator, and the theoretical framework**		
All M2M videos were stored on a free, secure, publicly available, and cloud-based video sharing service (YouTube).		
**5-ix) Describe use parameters**		
In addition to the M2M program, the intervention included behavioral coaching calls with a telecoach using Zoom videoconference software.		
**5-x) Clarify the level of human involvement**		
Participants were instructed to complete all videos within their playlist 3x/week, on non-consecutive days.		
**5-xi) Report any prompts/reminders used**		
In addition to the M2M program, the intervention included behavioral coaching calls with a telecoach using Zoom videoconference software.		
**5-xii) Describe any co-interventions (incl. training/support)**		
This is not applicable to the study.		
**6a) CONSORT: Completely defined pre-specified primary and secondary outcome measures, including how and when they were assessed**		

Eligibility criteria included: 1) a diagnosis of CP as determined by International Classification of Disease (ICD) codes from electronic medical records; 2) the ability to exercise with arms; 3) aged between 10-19 years (adolescent age range, as defined by the World Health Organization) [31]; 4) access to a Wi-Fi Internet connection in the home; and 5) a device that was capable of viewing Internet-video content (television, computer tablet, laptop, or desktop computer). Exclusion criteria included: 1) physically active (defined as >60 minutes per day of moderate-to-vigorous intensity exercise in a typical week) [32]; and 2) complete blindness or deafness.		
**6a-i) Online questionnaires: describe if they were validated for online use and apply CHERRIES items to describe how the questionnaires were designed/deployed**		
**6a-ii) Describe whether and how "use" (including intensity of use/dosage) was defined/measured/monitored**		
This is not relevant to the study.		
**6a-iii) Describe whether, how, and when qualitative feedback from participants was obtained**		
A telecoach monitored participant engagement by using the YouTube Analytics web-application.		
**6b) CONSORT: Any changes to trial outcomes after the trial commenced, with reasons**		
Screening, recruitment, and data collection procedures were conducted remotely.		
**7a) CONSORT: How sample size was determined**		
**7a-i) Describe whether and how expected attrition was taken into account when calculating the sample size**		
**7b) CONSORT: When applicable, explanation of any interim analyses and stopping guidelines**		
The primary outcome was pre to post changes in LTPA after the 4-week M2M intervention. At week 0 and week 4, LTPA was measured by the Children's Assessment of Participation and Enjoyment (CAPE) [40]... The secondary aim was to explore the potential program effects on perceived pain and fatigue.		
**8a) CONSORT: Method used to generate the random allocation sequence**		
This is not relevant to the study.		
**8b) CONSORT: Type of randomisation; details of any restriction (such as blocking and block size)**		
This is not applicable to the study.		
**9) CONSORT: Mechanism used to implement the random allocation sequence (such as sequentially numbered containers), describing any steps taken to conceal the sequence until interventions were assigned**		
After baseline assessments were completed and returned, the recruiting staff contacted the project statistician to determine a participant's allocation to a group.		
**10) CONSORT: Who generated the random allocation sequence, who enrolled participants, and who assigned participants to interventions**		
Participants were randomized into one of two groups (M2M or waitlist control) with a 1:1 allocation ratio using a permuted block randomization approach. Participants were stratified based on their functional level into either a standing (GMFCS levels I-III) or seated M2M program (GMFCS levels IV-V). The randomization sequence was generated a priori by the project statistician using a computer-generated random schedule in permuted blocks (SAS V.9.4).		
**11a) CONSORT: Blinding - If done, who was blinded after assignment to interventions (for example, participants, care providers, those assessing outcomes) and how**		
**11a-i) Specify who was blinded, and who wasn't**		
**11a-ii) Discuss e.g., whether participants knew which intervention was the "intervention of interest" and which one was the "comparator"**		
Only the statistician knew the randomization sequence (the investigators and interventionists were blinded to the randomization process).		
**11b) CONSORT: If relevant, description of the similarity of interventions**		
Only the statistician knew the randomization sequence (the investigators and interventionists were blinded to the randomization process). Given the nature of the intervention and control, it was not applicable to blind the participants or the telecoach during the intervention.		
**12a) CONSORT: Statistical methods used to compare groups for primary and secondary outcomes**		
This is not applicable to the study.		
**12a-i) Imputation techniques to deal with attrition / missing values**		
**12b) CONSORT: Methods for additional analyses, such as subgroup analyses and adjusted analyses**		
This is not relevant to the present study.		
RESULTS		
**13a) CONSORT: For each group, the numbers of participants who were randomly assigned, received intended treatment, and were analysed for the primary outcome**		
Minor clarifications were fixed throughout the manuscirpt.		
**13b) CONSORT: For each group, losses and exclusions after randomisation, together with reasons**		
This was not applicable to the study.		
**13b-i) Attrition diagram**		
**14a) CONSORT: Dates defining the periods of recruitment and follow-up**		
Out of 521 people contacted, 82 were screened, 59 were enrolled, 51 completed the intervention, and 49 completed the intervention and post CAPE assessment.		
**14a-i) Indicate if critical "secular events" fell into the study period**		
**14b) CONSORT: Why the trial ended or was stopped (early)**		
Participant flow data are displayed in Figure 1.		
**15) CONSORT: A table showing baseline demographic and clinical characteristics for each group**		
Recruitment occurred from September 2020 to September 2021.		
**15-i) Report demographics associated with digital divide issues**		
**16a) CONSORT: For each group, number of participants (denominator) included in each analysis and whether the analysis was by original assigned groups**		
**16-i) Report multiple "denominators" and provide definitions**		
**16-ii) Primary analysis should be intent-to-treat**		
This was not applicable to the study.		
**17a) CONSORT: For each primary and secondary outcome, results for each group, and the estimated effect size and its precision (such as 95% confidence interval)**		
Fourth, this project initially aimed to test an 8-week M2M program. Due to COVID-19 restrictions, we experienced a 6-month delay in university-related operations, which required us to shorten the intervention to complete the study within a 1.5-year period.		
**17a-i) Presentation of process outcomes such as metrics of use and intensity of use**		
**17b) CONSORT: For binary outcomes, presentation of both absolute and relative effect sizes is recommended**		
Participant characteristics are shown in Table 1.		
**18) CONSORT: Results of any other analyses performed, including subgroup analyses and adjusted analyses, distinguishing pre-specified from exploratory**		
ANCOVA revealed statistically significant between group differences in pre to post change score for CAPE-Intensity (F (1, 47)=5.63, p = 0.022, effect size=0.11) (a.k.a. volume of activity) and CAPE-APR-Intensity (F (1, 47)=8.76, p = 0.0048,effect size=0.17; a.k.a. volume of LTPA).		
**18-i) Subgroup analysis of comparing only users**		
**19) CONSORT: All important harms or unintended effects in each group**		
This is not relevant to the study.		
**19-i) Include privacy breaches, technical problems**		

**19-ii) Include qualitative feedback from participants or observations from staff/researchers**		
This was not applicable to the study.		
DISCUSSION		
**20) CONSORT: Trial limitations, addressing sources of potential bias, imprecision, multiplicity of analyses**		
**20-i) Typical limitations in ehealth trials**		
**21) CONSORT: Generalisability (external validity, applicability) of the trial findings**		
**21-i) Generalizability to other populations**		
**21-ii) Discuss if there were elements in the RCT that would be different in a routine application setting**		
Third, our sample was short of our target by 5 people.		
**22) CONSORT: Interpretation consistent with results, balancing benefits and harms, and considering other relevant evidence**		
**22-i) Restate study questions and summarize the answers suggested by the data, starting with primary outcomes and process outcomes (use)**		
**22-ii) Highlight unanswered new questions, suggest future research**		
Quantitative findings demonstrated that the M2M program resulted in a small increase (effect size=0.17) in the volume of LTPA performed by the individual in the home setting, compared with WC.		
Other information		
**23) CONSORT: Registration number and name of trial registry**		
This is not relevant to the study.		
**24) CONSORT: Where the full trial protocol can be accessed, if available**		
No adverse events (e.g., accident, injury, or condition related to the intervention) were reported by participants.		
**25) CONSORT: Sources of funding and other support (such as supply of drugs), role of funders**		
Trial Registration: ClinicalTrials.gov NCT04264390		
**X26-i) Comment on ethics committee approval**		
**x26-ii) Outline informed consent procedures**		
The study protocol received full approval by the University Institutional Review Board (IRB) for Human Use at the University of Alabama at Birmingham (IRB#300004608).		
**X26-iii) Safety and security procedures**		
They had two options to complete the assessments: 1) physically mailed documents that were in a large envelope containing the informed consent document and all outcome measures or questionnaires; or 2) signature of a digital consent document through a secure electronic database (REDCap)...		
**X27-i) State the relation of the study team towards the system being evaluated**		
