# Supplementary material for: Telehealth Movement-to-Music With Arm-Based Sprint-Intensity Interval Training to Improve Cardiometabolic Health and Cardiorespiratory Fitness in Children With Cerebral Palsy: Protocol for a Pilot Randomized Controlled Trial
Source: JMIR Res Protoc. 2024 Mar 5;13:e56499. doi: 10.2196/56499 (PMC10951837; doi:10.2196/56499)
Supplement: Multimedia Appendix 3 [file resprot_v13i1e56499_app3.pdf]

Author Note (1/19/24):

This document provides evidence of review for the proposed protocol for this randomized controlled trial. The document below was the original review by NIH grant reviewers. Numerous modifications to the study protocol were made to address the concerns of the reviewers, particularly insufficient detail provided for the human subjects protections and plans for protections. Examples of resultant changes from this original review include, but are not limited to, the addition of a medical alert device, AED, and CPR/AED certification of caregiver participants, as well as the addition of real-time supervision for all exercise sessions through telehealth monitoring.

SUMMARY STATEMENT

PROGRAM CONTACT: **(Privileged Communication)** ***Release Date:*** **10/31/2022**

[redacted] ***Revised Date:***

Application Number: **1 R21 HD109358-01A1**
Formerly: **1R21HD109358-01**

Principal Investigator

LAI, BYRON

Applicant Organization: UNIVERSITY OF ALABAMA AT BIRMINGHAM

Review Group: [redacted]

Meeting Date: **10/13/2022** [redacted]
Council: **JAN 2023**
Requested Start: **04/01/2023**

Dual IC(s): **NR**

Project Title: **Improving Cardiorespiratory Fitness and Cardiometabolic Health among Children with Physical Disabilities through Movement-to-Music Telehealth with Arm-based Sprint-Intensity Interval Training**

[redacted]

Human Subjects: **48-At time of award, restrictions will apply**
Animal Subjects: **10-No live vertebrate animals involved for competing appl.**
Gender: **1A-Both genders, scientifically acceptable**
Minority: **1A-Minorities and non-minorities, scientifically acceptable**
Age: **6A-Children and Adults, scientifically acceptable**

[redacted]

[redacted]

1R21HD109358-01A1 Lai, Byron

PROTECTION OF HUMAN SUBJECTS UNACCEPTABLE

RESUME AND SUMMARY OF DISCUSSION: The overall goal of this resubmission application is to pilot a newly revised movement-to-music (M2M) program to improve cardiorespiratory fitness and cardiometabolic health among children with cerebral palsy (CP) who have functional impairments. The panel agreed on the significance and innovation of the project seeking to address a critical gap in the field for scalable and inclusive exercise interventions for children with CP, which also has the potential to improve adherence. The investigative team has a track record of productivity, with a history of collaboration, and is situated in an excellent environment. The panel agreed the resubmission was highly responsive to previous critiques. Notable strengths of the approach include a robust study design. During the discussion, the panel remarked that some of the study components related to the role of music preferences in the intervention, the sprint exercise dose (4-second bouts), and the choice of seated sprint exercise (versus other modalities) to improve upon cardiovascular risk factors were not entirely well-justified. However, the panel members also acknowledged the pilot feasibility stage of the project, which attenuated these concerns. Overall, the panel concluded that the strengths of this highly significant application outweigh the minor weaknesses and the project's potential impact is high.

DESCRIPTION (provided by applicant): Due to alarmingly low rates of exercise participation, children with physical disabilities are at substantially high risk for cardiovascular disease (CVD), related conditions, and CVD mortality as they age into adulthood. Regular participation in aerobic exercise is an effective non-pharmaceutical method for preventing cardiovascular disease and metabolic syndrome, but effective modalities such as walking, running, and cycling are often not suitable for children with disabilities, who have impaired mobility. The growing availability of internet access and acceptance of telehealth (due to the coronavirus pandemic) create an unprecedented opportunity to engage large, underserved groups of children with disabilities in exercise behavior. However, ensuring that they maintain regular participation over periods of time that are necessary to elicit changes in cardiometabolic health will require enjoyable, accessible, and age-appropriate programs. This application proposes a pilot project that builds upon our recent feasibility work. The project will pilot an evidence-based, scale-up movement-to-music (M2M) program that has been newly revised for children with cerebral palsy who have functional impairments. The program includes three novel components: 1) pre-recorded videos that are stylized with visual graphics and enjoyable music to themes (e.g., superheroes, sports, pop music) that were requested by our feasibility participants; 2) arm-based routines with sprint-intensity interval training (SIT), based on principles from the latest exercise physiology research in the general population; and 3) replicable cloud-based tele-monitoring procedures. Participants will be recruited from a children's hospital and network of community organizations. A total of 50 children with cerebral palsy will be randomized to one of two groups: 12 weeks of SIT-M2M or 12-week waitlist control that undergoes habitual activity before receiving SIT-M2M. The SIT prescription will include 3 sessions per week of 30 repeated sequences of 4-s of maximal arm exercise, with active recovery periods (~20-min sessions with a warm-up and cool down period). This study will compare changes between groups in outcomes related to cardiovascular fitness and core indicators of cardiometabolic health, namely, body composition, body weight, high sensitivity C-reactive protein, hemoglobin A1c, triglycerides, cholesterol, and blood pressure. Additionally, the study will record implementation metrics that will inform the design of a follow-up trial. Study findings will inform the development of a convenient telehealth efficacy trial for implementation across various sites and settings to improve the health and well- being of children with disabilities who cannot walk or stand for prolonged periods of time.

PUBLIC HEALTH RELEVANCE: This home-based, telehealth trial will pilot the preliminary efficacy of a scalable sprint interval training program with enjoyable themes and music to improve cardiovascular fitness and cardiometabolic health among children with cerebral palsy. This project has the potential to address a large knowledge gap in the extant literature, because there are no widely accessible, evidence-based, enjoyable, and age-appropriate modalities for improving cardiovascular fitness or cardiometabolic health among children with disabilities who have mobility disabilities. The study findings will inform a larger, definitive efficacy study that can be easily implemented by researchers and health professionals across a variety of settings.

[redacted]

[redacted]

Overall Impact: This is a resubmission of an R21 from an early stage PI. The study proposed is a phase I pilot clinical trial designed to confirm feasibility and test the effectiveness of a telehealth-delivered exercise intervention consisting of movement to music, and arm-based sprint interval training, for adolescents with cerebral palsy. The application was highly responsive to previous reviewer comments, which has greatly strengthened the application. The PI and team are very knowledgeable about the literature in this area, and have identified a major limitation of previous interventions is lack of scalability and inclusivity of those with mobility disabilities. The intervention proposed is novel, and also addresses these identified limitations through the use of telehealth and use of seated exercise modalities. Thus, this project has potential for high impact on the field by increasing availability of exercise options for individuals with cerebral palsy, as traditional exercise modalities are not appropriate for those who have mobility disabilities. In addition, the study team has extensive experience doing intervention work in this and similar populations, and are well suited to carry out the proposed study. There are a few minor to moderate weaknesses in the approach that lessen the overall impact slightly. These include the manner in which compliance and fidelity is being assessed, the rationale for a volume of exercise lower than suggested thresholds for improving cardiorespiratory fitness, and uncertainty that the desired intensity can be achieved in 4 second bouts. However, in spite of these limitations, the project has merit and is worth doing, and the team and environment are well-poised to execute the study. Overall if successful, the study has potential for high impact, with some weaknesses.

1. Significance:

Strengths

- A major limitation of previous interventions among individuals with cerebral palsy is lack of scalability and inclusivity of those with mobility disabilities. The proposed intervention has potential to addresses these identified limitations by using telehealth and arm-based exercise training which can be done while seated. Thus, this project has potential for high impact on the field by increasing availability of exercise options for individuals with cerebral palsy who have mobility disabilities.

Weaknesses

- Not entirely clear why sprint interval training was selected rather than other modalities that have also been shown to increase cardiorespiratory fitness.

2. Investigator(s):

Strengths

- PI has relevant expertise conducting studies for adults with disabilities and specifically a small trial of M2M with adolescents with cerebral palsy. Rimmer – senior colleague with extensive experience in telehealth and PA interventions for individuals with disabilities.
- Biostatistician added with relevant experience

Weaknesses

- None noted by reviewer

3. Innovation:

Strengths

- Adding arm-based sprint interval training to movement to music protocol is highly novel.

Weaknesses

- None noted by reviewer

4. Approach:

Strengths

- The outcomes included in feasibility assessment and objectively measured cardiorespiratory fitness.
- Movement to music element of the intervention is well developed and tested.
- The intervention has been designed with scalability and long-term sustainability of the program (e.g., enjoyment of the activities) in mind.

Weaknesses

- The previous study described enrolled *N*=58 in 1.5 years, but the intervention was only 1 month vs. the proposed 3-month (12 week) intervention. This raises some questions about the feasibility of recruiting this sample size (minor).
- The sprint interval training protocol is 30x4 second bouts. However, it is perplexing that the preliminary data from the case study presented used 3-minutes of high intensity exercise. This raises questions about whether 4 seconds would be enough to elicit the desired intensity, and thus sufficient overload to stimulate improvements in cardiorespiratory fitness. (moderate)
- Exploring cardiometabolic outcomes seems ambitious since it is unknown if the mediator of these outcomes – cardiorespiratory fitness - will improve. Further only one similar previous study has demonstrated improvements in cholesterol, and this was after 6-months. Thus there is a major bias toward null for this aim. (moderate)
- Proposing to do assessments in-person at a facility does not overcome the stated barriers of geographic distance, and lack of transportation. (minor)

- The investigators present evidence to suggest that 20 minutes of MVPA twice per week for 16 week is insufficient to observe meaningful changes in cardiometabolic health, which is contradictory to the even smaller exercise dose and duration planned in the proposed intervention. (moderate)
- HR monitoring would enhance the fidelity to exercise intensity, particularly in the unsupervised sessions. There is a high possibility that the instructor’s motivational strategies could play a role in participant’s adherence to the intensity, thus it will be unclear whether intensity is matched during supervised vs. unsupervised session, which is very important for informing next steps, which are planned to be fully unsupervised. (moderate)

5. Environment:

Strengths

- Excellent facilities, resources and equipment available to support all aspects of the proposed study.

Weaknesses

- None noted by reviewer

Study Timeline:

Strengths

- Time appropriately allocated to study activities

Weaknesses

- None noted by reviewer

Protections for Human Subjects:

Acceptable Risks and/or Adequate Protections

Data and Safety Monitoring Plan (Applicable for Clinical Trials Only):

Acceptable

Inclusion Plans:

- Sex/Gender: Distribution justified scientifically
- Race/Ethnicity: Distribution justified scientifically
- For NIH-Defined Phase III trials, Plans for valid design and analysis: Not applicable
- Inclusion/Exclusion Based on Age: Distribution justified scientifically

Vertebrate Animals:

Not Applicable (No Vertebrate Animals)

Biohazards:

Not Applicable (No Biohazards)

Resubmission:

- Applicants were responsive to previous critiques

Resource Sharing Plans:

Acceptable

Budget and Period of Support:

Recommend as Requested

[redacted]

[redacted]

Overall Impact: This revised R21 proposes to test a 12-week home-based SIT-M2M program on cardiorespiratory fitness compared to wait list control among 50 youth (6-19yrs) with cerebral palsy. The intervention is virtual/remote delivered, child themed, and provides interval training to enhance CVF. Overall, the investigators were responsive to the prior review. This is a strong application led by an experienced team of investigators. The focus on CwCP, the design of a remote intervention tailored for youth and enhancing CVF are all major strengths. There are no identifiable weaknesses. Thus, the proposed study by this investigative team has a high probability of success and strong impact on the field.

1. Significance:

Strengths

- The focus on CwCP is an important population to address CVF
- The design of the remote delivered intervention, with videos themed to appeal to youth, are great ideas and likely to appeal to the target population to keep them engaged. Additionally, if successful, this platform can be easily scaled for broader population impact.
- The use of SIT is well justified and likely to lead to marked improvement in the proposed outcomes

Weaknesses

- None noted

2. Investigator(s):

Strengths

- Very strong team with extensive experience in this population – these are the right people to conduct this type of study

Weaknesses

- None noted

3. Innovation:

Strengths

- Tailoring the videos to appeal to youth and remotely delivering really can increase adherence and reduce barriers to participation – all key factors in receiving sufficient dosages

Weaknesses

- None noted

4. Approach:

Strengths

- Randomized design
- Remote delivery and process evaluation
- Measures are appropriate
- Intervention is based on sound scientific rationale
- Target population is important

Weaknesses

- None noted

5. Environment:

Strengths

- This is a very strong environment with a history of working with this population

Weaknesses

- None noted.

Study Timeline:

Strengths

- Sufficiently detailed

Weaknesses

- None noted by reviewer

Protections for Human Subjects:

Acceptable Risks and/or Adequate Protections

Data and Safety Monitoring Plan (Applicable for Clinical Trials Only):

Acceptable

Inclusion Plans:

- Sex/Gender: Distribution justified scientifically
- Race/Ethnicity: Distribution justified scientifically
- For NIH-Defined Phase III trials, Plans for valid design and analysis: Not applicable
- Inclusion/Exclusion Based on Age: Distribution justified scientifically

Vertebrate Animals:
Not Applicable (No Vertebrate Animals)

Biohazards:
Not Applicable (No Biohazards)

Resubmission:
Overall, the investigators were responsive to the prior review.

Resource Sharing Plans:
Acceptable

Budget and Period of Support:
Recommend as Requested

[redacted]

[redacted]

Overall Impact: This is a very strong proposal that seeks to explore the impact of sprint-intensity interval training on cardiovascular fitness in children with cerebral palsy. Understanding how we can improve cardiovascular fitness in CwCP is important scientifically and from a public health perspective. There is a strong investigative team in place who are experts in this area. The combination of testing a sprint-intensity interval training approach with an eye toward making it enjoyable for children is innovative. Using teleconference software will increase the probability of reaching recruitment goals and eventually scaling the intervention. There is also a very strong environment in place to support the work. Overall, this is an impactful application that has the potential to lead to improved cardiovascular health in CwCP.

1. Significance:

Strengths

- Understanding how we can improve cardiovascular fitness in CwCP is important scientifically and from a public health perspective.
- Review of the literature is rigorous and points to the proposed intervention.
- The research will address both the potential clinical impact as well as a scientific knowledge gap.
- Addressing feasibility (aim 3) is important at this phase of the work.

Weaknesses

- The impact of attention is not controlled for, but for this initial pilot study it does not appear to be a necessary component.

2. Investigator(s):

Strengths

- Strong team with both senior and junior members.
- Team has a great deal of experience in this area.
- Includes statistical support.

Weaknesses

- No appreciable weaknesses.

3. Innovation:

Strengths

- The combination of testing a sprint-intensity interval training approach with an eye toward making it enjoyable for children (videos) is innovative.

Weaknesses

- Could be greater incorporation of innovative theory.

4. Approach:

Strengths

- RCT design incorporated.
- Intent to treat principles are incorporated.
- The inclusion of video conference technologies that will enhance future scalability.
- Determining the initial impact of SIT interventions on cardiovascular risk factors will add to our understanding of exercise effects in CwCP.
- Addressing feasibility of the intervention within the design.

Weaknesses

- Lack of incorporation of behavior change theory.

5. Environment:

Strengths

- A very strong environment that will be supportive of the work.

Weaknesses

- No appreciable weaknesses.

Study Timeline:

Strengths

- A study timeline is included.

Weaknesses

- Lack of detail regarding study related activities and benchmarks (e.g., IRB approval, hiring of personnel).

Protections for Human Subjects:

Acceptable Risks and/or Adequate Protections

- Protections against risk are in place.

Data and Safety Monitoring Plan (Applicable for Clinical Trials Only):

Acceptable

- A DSMP is in place.

Inclusion Plans:

- Sex/Gender: Distribution justified scientifically
- Race/Ethnicity: Distribution justified scientifically
- For NIH-Defined Phase III trials, Plans for valid design and analysis: Not applicable
- Inclusion/Exclusion Based on Age: Distribution justified scientifically
- Distributions are well-justified.

Vertebrate Animals:

Not Applicable (No Vertebrate Animals)

Biohazards:

Not Applicable (No Biohazards)

Resubmission:

Acceptable

Resource Sharing Plans:

Acceptable

Budget and Period of Support:

Recommend as Requested

[redacted]

[redacted]

Overall Impact: This R21 application proposes an ambitious study examining a 12-week exercise intervention program for children and adolescents with CP. The significance and innovation are very high, as there is very little research on exercise interventions for children with CP, the approach for the proposed intervention is scalable, and the study design is rigorous for a pilot study; existing research in this field is limited by much smaller sample sizes than the researchers propose (50) as well as challenges related to scalability (i.e., most existing research has looked at interventions that are administered in person and require substantial support for implementation in terms of personnel, etc.). Additional strengths included a very strong team and an outstanding environment for this type of research. The investigators have used patient feedback to design the intervention and inform the selection of themes, though it's not clear if patient feedback informed the selection of music. The role and impact of music in the proposed intervention is under-developed, and it appears that it is included simply as a technique to improve motivation/engagement, without adequate consideration of the emotion and impact of various genres of music. The data collection plan for physiological outcomes is rigorous, and the investigators have added a brief and commonly used questionnaire to obtain feedback from participants related to engagement and enjoyment of the exercise. However, this third aim remains under-developed, and there are no described plans to obtain qualitative or quantitative data that would specifically address features related to the design and potential improvement of the intervention (e.g., music preferences and relatability of the chosen themes to children with various gender identities, a very broad age range, and diverse interests). This information will be critical to the success of the future R01 as well as the refinement of the intervention protocol for youth of various ages. The recruitment target is ambitious, but the investigators have made a compelling argument for their ability to access the patients needed for the trial. The application did not adequately address protection against risks; the human subjects plan noted, appropriately, that exercise risks include fatigue, soreness, dizziness, nausea, and even risk of death. As this exercise will take place in participant's homes, the human subjects plan did not adequately describe how risks would be monitored and how appropriate response to adverse effects of exercise might be conducted (i.e., will it be a requirement that a caregiver is present during the exercise? Will caregivers be given education and training about monitoring participants and knowing when/how to contact investigators or seek medical or emergency help?) Overall, in spite of a great deal of enthusiasm for this application, the overall impact score falls in the moderate range as concerns about human subjects and the under-development of the third aim weaken the potential overall impact of the study.

Protections for Human Subjects:

Unacceptable Risks and/or Inadequate Protections

- The application also did not adequately address protection against risks; the human subjects plan noted, appropriately, that exercise risks include fatigue, soreness, dizziness, nausea, and even risk of death. As this exercise will take place in participant's homes, the human subjects

plan did not adequately describe how this would be monitored and how appropriate response to adverse effects of exercise might be conducted (i.e., will it be a requirement that a caregiver is present during the exercise? Will caregivers be given education and training about monitoring participants and knowing when/how to contact investigators or seek help?)

Data and Safety Monitoring Plan (Applicable for Clinical Trials Only):

Acceptable

Inclusion Plans:

- Sex/Gender: Distribution justified scientifically
- Race/Ethnicity: Distribution justified scientifically
- For NIH-Defined Phase III trials, Plans for valid design and analysis: Not applicable
- Inclusion/Exclusion Based on Age: Distribution justified scientifically

Vertebrate Animals:

Not Applicable (No Vertebrate Animals)

Biohazards:

Not Applicable (No Biohazards)

Resource Sharing Plans:

Acceptable

Budget and Period of Support:

Recommend as Requested

THE FOLLOWING SECTIONS WERE PREPARED BY THE SCIENTIFIC REVIEW OFFICER TO SUMMARIZE THE OUTCOME OF DISCUSSIONS OF THE REVIEW COMMITTEE, OR REVIEWERS' WRITTEN CRITIQUES, ON THE FOLLOWING ISSUES:

PROTECTION OF HUMAN SUBJECTS: UNACCEPTABLE

The project did not adequately address the protection of human subjects related to exercise risk in the home and additional description for monitoring adverse effects is warranted.

INCLUSION OF WOMEN PLAN: ACCEPTABLE

INCLUSION OF MINORITIES PLAN: ACCEPTABLE

INCLUSION ACROSS THE LIFESPAN: ACCEPTABLE

COMMITTEE BUDGET RECOMMENDATIONS: The budget was recommended as requested.

[redacted]

[redacted]

[redacted]
